# A Quantitative Analysis of Growth and Size Regulation in *Manduca sexta*: The Physiological Basis of Variation in Size and Age at Metamorphosis

**DOI:** 10.1371/journal.pone.0127988

**Published:** 2015-05-26

**Authors:** Laura W. Grunert, Jameson W. Clarke, Chaarushi Ahuja, Harish Eswaran, H. Frederik Nijhout

**Affiliations:** Department of Biology, Duke University, Durham, NC 27708, United States of America; Natural Resources Canada, CANADA

## Abstract

Body size and development time are important life history traits because they are often highly correlated with fitness. Although the developmental mechanisms that control growth have been well studied, the mechanisms that control how a species-characteristic body size is achieved remain poorly understood. In insects adult body size is determined by the number of larval molts, the size increment at each molt, and the mechanism that determines during which instar larval growth will stop. Adult insects do not grow, so the size at which a larva stops growing determines adult body size. Here we develop a quantitative understanding of the kinetics of growth throughout larval life of *Manduca sexta*, under different conditions of nutrition and temperature, and for genetic strains with different adult body sizes. We show that the generally accepted view that the size increment at each molt is constant (Dyar’s Rule) is systematically violated: there is actually a progressive increase in the size increment from instar to instar that is independent of temperature. In addition, the mass-specific growth rate declines throughout the growth phase in a temperature-dependent manner. We show that growth within an instar follows a truncated Gompertz trajectory. The critical weight, which determines when in an instar a molt will occur, and the threshold size, which determines which instar is the last, are different in genetic strains with different adult body sizes. Under nutrient and temperature stress *Manduca* has a variable number of larval instars and we show that this is due to the fact that more molts at smaller increments are taken before threshold size is reached. We test whether the new insight into the kinetics of growth and size determination are sufficient to explain body size and development time through a mathematical model that incorporates our quantitative findings.

## Introduction

Size and age at maturity are important life history traits. Body size can affect reproductive capacity [1–3] and development time can affect generation time [4], so both can affect the rate of increase of a population or the rate at which favorable genes can spread. Body size is also one of the defining characteristics of a species. Although most species vary little around a species-characteristic mean, selection experiments have shown that body size responds readily to selection [5–8], and in insects laboratory experiments have shown that body size is plastic and can be made to vary over a broad range. In *Manduca sexta*, for instance, the typical maximal larval mass is 11.3 +/- 1.5 grams (StDev), and it has been possible to select for strains that vary in body size (mean maximal larval mass) from 5.9 to 16.1 grams. Nutrient manipulation can induce an additional two-fold variation in pupal mass [9,10]. Such observations suggest that the small amount of phenotypic variation we observe under natural conditions must be due to the fact that body size is robust to normally-occurring genetic and environmental variation. This robustness must arise from the developmental and physiological mechanisms that regulate body size.

During the past decade the genetic and physiological mechanisms that control growth, body size and development time in insects have become increasingly well understood [11,12]. Nutrient-dependent growth is stimulated by hormones and growth factors. In insects, insulin-like growth factors and the steroid hormone ecdysone are the primary regulators of cell tissue and somatic growth [11]. Disruption of insulin signaling in *Drosophila* leads to dwarfed animals [13,14]. Ecdysone, by contrast, is required for normal tissue growth at low concentrations, but at high concentrations ecdysone stops growth and leads to tissue differentiation [15].

Pupae and adults insects do not grow, so adult body size is determined by the size at which a larva begins the metamorphic molt to the pupa. This molt is triggered by a pulse of ecdysone secretion. Thus the time and body size at which this ecdysone pulse occurs determines the age and size at metamorphosis. Body size regulation at the physiological level depends on the decision pathway that leads to the secretion of ecdysone, and this pathway is affected by both genetic and environmental variables. Previous work has shown that it is possible to accurately predict the final size of a larva based on three empirically measurable parameters of the last larval instar: the growth rate, the critical weight, and the duration of the terminal growth phase [10]. The predictive model is accurate over a two-fold range in final body mass for several genetic strains with different body sizes.

Most of what we know about the regulation of growth and size comes from studies of last-instar larvae. The rationale is that in systems that grow approximately exponentially, most of the mass accumulates in the last larval instar. In *Manduca*, for instance, about 90% of growth in mass occurs during the fifth (final) instar [10,16]. The assumption has been that growth in earlier instars is just like that in the last larval instar, just scaled down [17]. Moreover, it has been assumed implicitly that because most of the growth occurs in the last larval instar that variation in growth in earlier instars is unlikely to have a significant effect on growth in the final instar and on variation in final body size.

These assumptions are, however, may or may not be correct. Although under optimal conditions and in the laboratory *Manduca* invariably has five larval instars; in the field and when reared on host plants that provide inadequate nutrition, *Manduca* can have supernumerary larval instars [18–20]. A variable number of larval instars is actually quite common in insects, but the underlying growth kinetics that gives rise to such variation has never been studied in any detail. The predictive model of body size regulation in *Manduca* growth [10] is adequate for 5th instar larvae growing under near optimal conditions, but says nothing about whether and how variation in growth of earlier instars affects the growth of the fifth larval instar, and has no information about the causes and consequences of the mechanism that control the number of larval instars.

In this paper we describe a detailed analysis of the kinetics of growth of *Manduca* under a range of nutritional and temperature conditions that cause variation in the number of larval instars and in the time and size at metamorphosis. This analysis revealed several unexpected features, such as a systematic violation of Dyar’s Rule under all growth conditions. The growth kinetics of larvae change systematically from instar to instar, as does the critical size at which each successive molt is initiated. And in slow-growing larvae, molts are not cued by reaching a critical size but appear to occur in a clock-like fashion. We show that certain combinations of temperature and nutrition can cause larvae to undergo as many as 8 larval instars and specify the conditions under which supernumerary instars are produced.

In order to verify our new understanding of growth and size regulation we developed a mathematical model that incorporates the new findings described in this paper finding and that accurately reproduces growth trajectories under a variety of temperatures and nutritional conditions.

## Methods

Larvae of *Manduca sexta* were reared on an artificial diet described in [16] in temperature-controlled rooms under a photoperiod of 16 hours light and 8 hours darkness. Reduction in the nutritive value of the diet was done in two ways as indicated in the text: (1) by replacing a percentage of all nutrients (except vitamins, cholesterol and antibiotics) with an equal weight of non-nutritive cellulose, or (2) by replacing only a fraction of the protein and amino acid-containing ingredients (wheat germ, yeast extract and casein hydrolysate) with an equal weight of non-nutritive cellulose. All temperatures were maintained within 0.5 degrees Celsius of their indicated values. Weights were recorded daily using a Mettler AE50 analytical balance with a resolution of 0.1 milligrams for weight below 1 gram and a Sartorius 3719 analytical balance with a resolution of 10 milligrams for weight above 1 gram. Larvae were weighed daily at 5–7 hours after lights-on. Development time was determined as the period from hatching to the end of the growth phase, which is the day on which larvae entered the wandering stage. Wandering begins in the dark phase, about 2–4 hours before lights-on. Because wandering larvae lose weight for several days, the peak weight of the larva was taken as the weight on the day prior to entry into the wandering stage. Juvenile hormone treatment used methoprene, a juvenile hormone analog. Methoprene was dissolved in acetone and applied topically to the dorsum in a 10 microliter dose, containing 5 micrograms of methoprene. Threshold size was determined from larvae growing on nutrient-deficient diets. Such larvae varied greatly in mass at each molt. Larvae were scored as 1 if the instar they molted to was the final larval instar and as zero if not. The moving average of 6 larvae was used to determine the value of the threshold mass at which >50% of larvae (mean score = 0.5) entered the last larval instar. The genetic strains used were those described previously [21].

## Results

### Size increment at molting: Dyar’s Rule and stepwise increase

**Fig 1:**
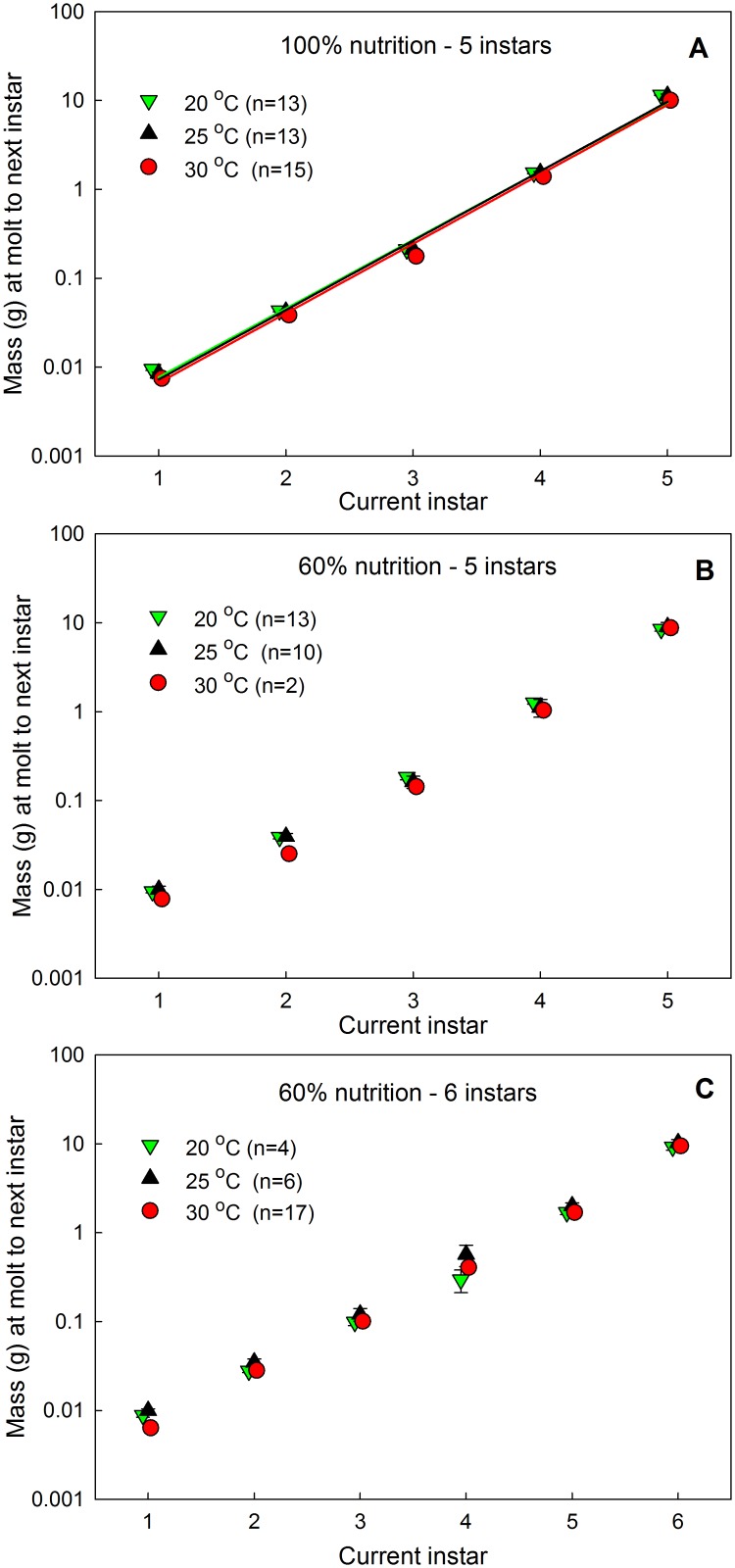
Dyar relationships between instar number and mass at the end of that instar. (A) Wild-type strain at normal nutrition and different temperatures. Exponential regressions are shown and given by: for 20°C, 0.0013e^1.778^ r^2^ = 0.995; for 25°C, 0.0012e^1.798^s r^2^ = 0.997; for 30°C, 0.001e^1.799^ r^2^ = 0.996. (B) Wild-type strain growing on low nutrient diet and different temperatures undergoing 5 larval instars. (C) Wild-type strain growing on low nutrient diet and different temperatures undergoing 6 larval instars. Bars are SEMs and are mostly smaller than the symbols.

When insects grow under constant conditions, the dimensions of their exoskeleton generally increase by a constant factor at each molt. This results in a geometric progression of size from instar to instar. This constancy of the size increment is embodied in Dyar’s Rule [22–24] and appears to hold not only for linear measures of the exoskeleton, but also for body mass at the time of molting [10]. The general expression of Dyar’s Rule is: size at be beginning of instar = a * e ^d*number of the instar^, where *d* is the Dyar coefficient; for dimensions that change in the course of an instar, such as body mass, the mass at either the beginning or at the end of the instar can be used. A Dyar coefficient of 1.72, for instance, indicates that at each molt the initial mass of the larva increases by *e* ^1.72^ = 5.6-*fold*. Fig 1A shows the Dyar relationship for mass at the time of molting for wild-type *Manduca* larvae feeding on a ‘normal’ diet, at three different temperatures. Tables 1 and 2 show the values for the Dyar coefficients for *Manduca* larvae growing at different temperatures and nutrient conditions and for 5 genetic strains of different final body sizes and larval development times. The Dyar coefficient appears to be independent of genetic background, temperature, and nutrition, but only for larvae undergoing 5 larval instars. At reduced nutrition *Manduca* larvae can undergo supernumerary instars, particularly when growing at elevated temperatures (see below). We have recorded individuals with as many as 8 larval instars. Larvae that grow with smaller increments at each molt have supernumerary instars. We found that Dyar coefficients of 1.6–1.8 lead to 5 instars, coefficients of 1.2–1.5 lead to 6 instars, and coefficients of 0.9–1.1 lead to 7 and 8 instars.

**Table 1:** Dyar coefficients (wild type) at different temperature and nutrient conditions.

Temperature °C	Diet %	Instars	Coefficient	(Std Err)	n
20	100	5	1.685	(0.0065)	13
25	100	5	1.720	(0.0053)	13
30	100	5	1.722	(0.0081)	15
25	60	5	1.711	(0.0163)	10
25	60	6	1.423	(0.0387)	8
30	60	5	1.273	(0.043)	2
30	60	6	1.168	(0.020)	17
30	60	7	1.015	(0.067)	2

**Table 2:** Dyar coefficients for genetic strains with different body sizes and development times (at 25°C and 100% diet).

Strain	n	Dyar coefficient	Maximum weight (grams (SEM))	Development time (days (SEM))
Dwarf (small/fast)	16	1.662	9.4 (0.22)	16.8 (0.07)
Giant (large/fast)	10	1.797	12.8 (0.42)	17.5 (0.05)
Wild-type (control)	13	1.720	10.8 (0.31)	19.1 (0.11)

Note: Dyar coefficients seem to be the same at all temperatures, diet qualities and body-size/development-time strains (c.f. Table 1). The biggest differences are in animals that have supernumerary instars.

**Fig 2:**
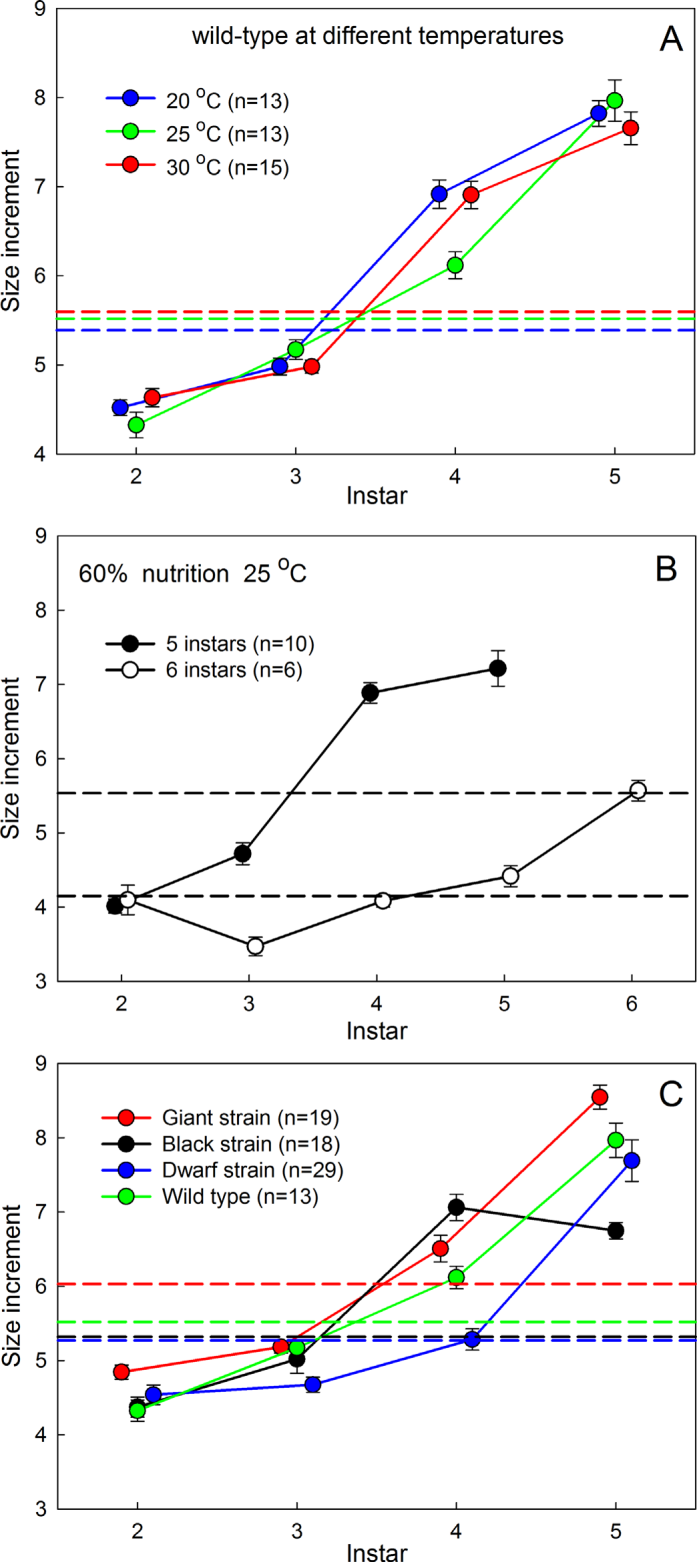
Size increments at each molt. (A) The wild-type strain at different temperatures. (B) The wild-type strain at 25°C under reduced nutrient condition undergoing either 5 or 6 larval and instars. (C) Three genetic strains (and wild type from A) with different adult body sizes at 25°C. Dashed lines show the corresponding (constant) values of the size increments predicted by the Dyar coefficient if Dyar’s Rule had applied. Bars are SEMs.

Dyar’s Rule assumes that there is a constant and identical increase in dimensions at each molt, and this view is reinforced by the apparent excellent fit of exponential regressions such as those illustrated in Fig 1A; the high r^2^ values for the regressions would generally be taken as evidence for a good fit. We found, however, that there are significant and systematic deviations from the apparent constancy of the size increase: the size increment actually increases from instar to instar (Fig 2). This deviation is difficult to see when size is plotted on a logarithmic axis, and thus might seem minor, but the difference in size increment between instars is highly significant (cf Figs 1 and 2). If Dyar’s Rule holds true, the size increment would be identical from instar to instar. In Fig 2 the horizontal dashed lines indicate the expected values of the size increment based on the Dyar coefficient obtained by exponential regression as shown in Tables 1 and 2. The value of this theoretical constant size increment is given by *ln(Dyar coefficient)* and corresponds to the geometric mean of actual size increments. Temperature has no effect on the size increment in successive instars (Fig 2A). Nutrition, by contrast, does affect the pattern of size increases (Fig 2B). Larvae reared on the 60% diet grew with smaller average size increments, as suggested by the lower Dyar coefficient in Table 1. For larvae that had 5 instars, the size increments followed the same pattern as those shown in Fig 2A and 2B. Larvae fed on the 60% diet that had 6 instars grew with much lower size increments (Fig 2B). The difference in size increment between larvae that will have 5 or 6 instars is evident as early as the 3rd instar.

We also measured the size increments in three genetic strains of *Manduca* that have different final body sizes (Fig 2C). The Black strain, which has a smaller body size than wild-type, had a much smaller size increment in the last larval instar than expected. The Giant and Dwarf strain had larger and smaller size increments, respectively, than the wild-type strain throughout larval life.

Although larvae reared on a nutrient-deficient diet appear to segregate into two cohorts with 5 and 6 instars, respectively (Fig 2B), in reality there is a rather broad range of individually variable size increments at each molt. Overall, larvae with 6 instars are in the lower end of the distributions of size increments, but the two cohorts do not become distinct until the 4th instar when the size increments of larvae that will have 5 instars increase but those that will have 6 instars remain small.

### Threshold size and the number of instars

Although under standard laboratory conditions *Manduca* invariably has 5 larval instars, in the field and under nutrient restriction in the laboratory, the number of instars can be variable [19,20]. We studied the effect of two kinds of nutrient restriction on the growth rate and duration of the larval stage at different temperatures. A restriction of only the protein component of the diet to 60% of normal (the missing bulk being made up by non-nutritive cellulose) reduced the growth rate and increased the duration of the larval stage (Table 3). The duration of the larval feeding period ranged from 12 to 45 days depending on nutrition, temperature and number of instars.

**Table 3:** Duration of larval development as a function of nutrition and temperature.

Nutrition	Instars	Temperature		
		20°C	25°C	30°C
normal diet	5	29 (2–33)*	18 (17–20)	13 (12–14)
60% diet	5	38 (34–45)	23 (22–26)	16 (15–17)
	6	43 (41–45)	27 (23–36)	18 (16–21)

* days (range)

**Fig 3:**
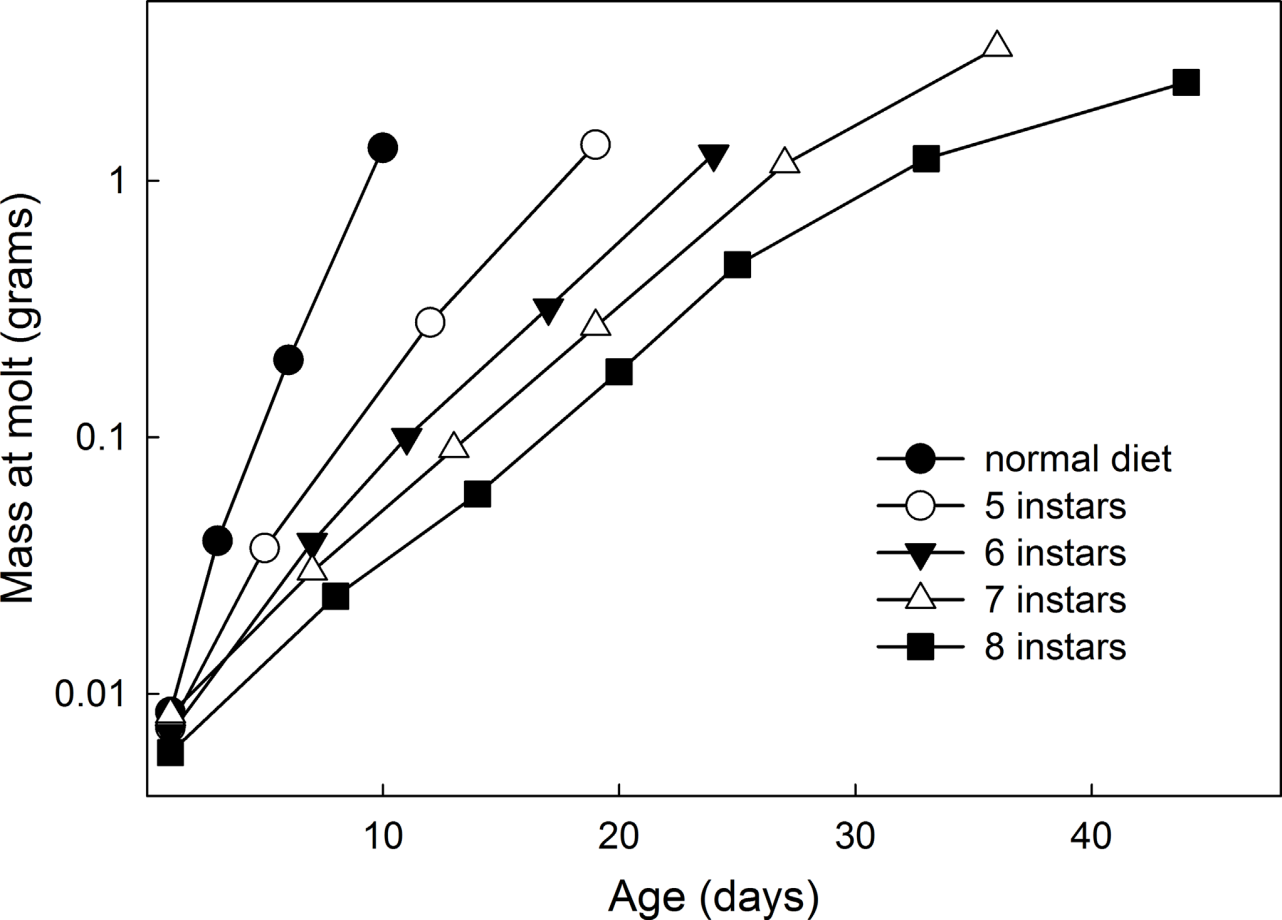
Growth trajectories for individual larvae growing on a low nutrient diet that had 5–8 instars. Each symbol represents time and mass at the molt to a new instar.

Reduction of all nutrients (except vitamins and cholesterol) in the diet to 60% of normal resulted in great variability of growth rate and an increase in the number of larval instars. The slowest growing larvae underwent as many as 8 larval instars (Fig 3). The fraction of larvae undergoing supernumerary instars on a low-nutrient diet increased with temperature (Table 4).

**Table 4:** Fraction of larvae making supernumerary instars.

Diet	Instars	Temperature (°C)		
		20	25	30
60% protein	5	0.80 (n = 12)	0.64 (n = 9)	0.11 (n = 2)
	6	0.20 (n = 3)	0.36 (n = 5)	0.89 (n = 17)
60% total nutrients	5		0.29 (n = 4)	
	6		0.21 (n = 3)	
	7		0.36 (n = 5)	
	8		0.14 (n = 2)	

Effect of diet and temperature.

**Fig 4:**
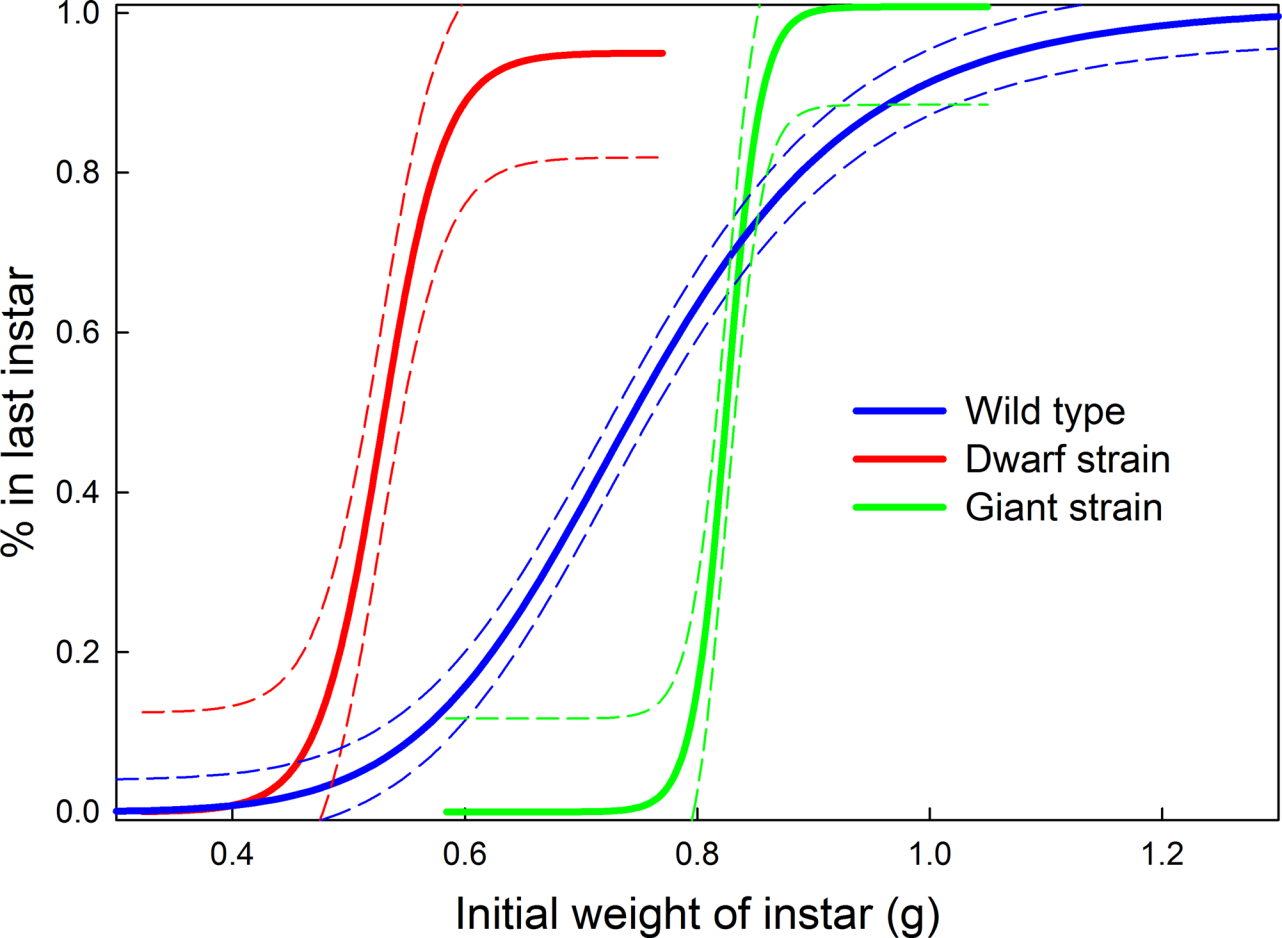
Threshold sizes for metamorphosis. Illustrating 3 genetic strains with different body sizes. Threshold size is the initial mass of the instar above which a larva is in the last instar. Wild-type: 6-point moving average of 189 individuals. Giant: 6-point moving average of 49 individuals. Dwarf: 6-point moving average of 62 individuals. Dashed lines are 95% prediction bands.

Supernumerary instars can appear when early instar larvae molt at smaller sizes than normal. Lepidopteran larvae become physiologically and developmentally committed to metamorphose at the time of the molt to the last larval instar [25–27]. The number of preceding larval instars is not a fixed genetic property but is strongly influenced by environment. The commitment to metamorphose is not determined by the number of the instar but by a property called the “threshold size,” which is operationally measured as the mass or head capsule width of the larva at the time of the molt [19,20]. Larvae above the threshold size are in the last larval instar, those below are not. We determined the threshold size of our wild-type strain and of our selected Giant and Dwarf strains (Fig 4). The transition to the final larval instars occurred over a broader range of sizes in the wild-type strain than in the two selected strains. Taking the weight at which 50% of larvae transition to the last larval instar as the threshold size, then for the wild-type it was 0.75 g, and for the Giant and Dwarf stains it was 0.83 g and 0.53 g, respectively. Thus the threshold size is a property whose value and accuracy can evolve under selection.

Smaller body size at a molt can have several causes. Within an instar, larvae are committed to molt when they pass the critical weight. In the final instar larvae of *Manduca* this occurs roughly half way through the instar, at about half the weight to which the larva will eventually grow [16,28]. The time between passing the critical weight and the molt is fixed and therefore if larvae are malnourished and grow slowly after they have passed the critical weight they will molt at a smaller than normal size. The size at which a larva molts in turn sets the critical weight for the next instar [17], therefore under malnutrition there is a progressive reduction in the critical weights and in the sizes at molt from instar to instar. As a consequence (as also illustrated in Fig 2B), slow growing larvae molt at smaller size increments and may take many more molts to reach the threshold size.

The tendency to produce supernumerary instars at low nutrition and high temperature (Table 4) can be explained by the fact that at high temperatures development is faster and the terminal growth phase—the period between attaining the critical weight and the molt—is foreshortened [9,29]. As a result, less mass is accumulated in spite of the fact that growth is faster.

### Growth within an instar is a truncated Gompertz trajectory

**Fig 5:**
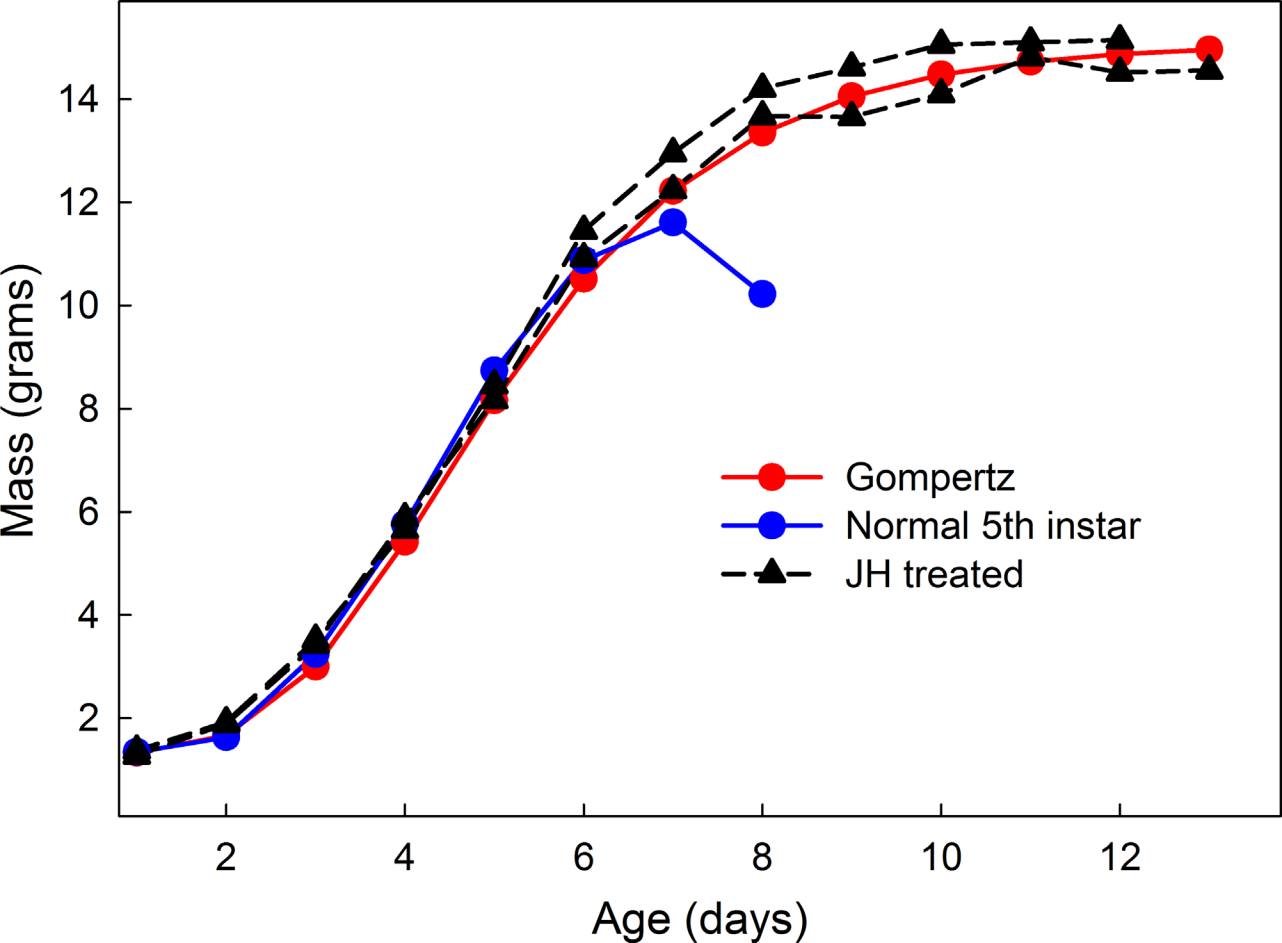
Growth trajectories. Two JH-treated larvae (black) illustrate growth uninterrupted by ecdysone secretion. The Gompertz curve (red) was fitted by non-linear regression. Blue line is a typical 5th instar wild-type growth trajectory.

Within an instar, growth is initially exponential but the exponent gradually declines [10], so that the overall growth trajectory is sigmoidal. Growth stops well before the sigmoidal asymptote is reached. Growth stops when a pulse of ecdysone is secreted, which causes a larva to stop feeding and initiate the next molt or, in the last larval instar, causes entry into the wandering stage in preparation for pupation [30]. This ecdysone pulse occurs well before a larva reaches its maximum potential size. In the last larval instar it is possible to prevent ecdysone secretion by topical application of juvenile hormone [10,28]. Larvae that are treated with juvenile hormone (JH) grow almost 50% percent larger than they normally would. Their growth gradually tapers off and although they may live and continue to feed for a long time even though there is no further growth. Examples of JH-treated growth curves are shown in Fig 5.

Growth curves of JH-treated larvae are well-fit by the Gompertz equation (Fig 5). The Gompertz equation describes exponential growth ($\frac{dM}{dt}\;=\;k*M$) with an exponential decline of the growth exponent ($\frac{dk}{dt}\;=\;-c*k$). The solution to these two differential equations is

$$M(t)=w_{i}+a*e^{-\frac{d}{c}*e^{-c_{*}t}},$$ (1)

where *M(t)* is the mass at time *t*, *a* is the maximum size (the upper asymptote) when *t* is large, *c* is the decay constant of the growth exponent and corresponds to the mass-specific growth rate at the inflection point, *d* is the mass-specific growth rate when *t* is zero and *w*_*i*_ is the initial weight. The three parameters (a, c and d) define the growth trajectory and can be obtained by non-linear regression on empirical growth curves.

The Gompertz curve has an inflection point at at $\frac{a}{\text{exp}\left(1\right)}+w_{i}$, which, we found, closely corresponds to the critical weight (Fig 5 and see also [10]). We found that the asymptotic mass, *a*, in JH-treated last-instar larvae is a constant multiple of the initial mass of the instar (*a* = *w*_i_*11.7±0.2 *SEM*) across a large range of body sizes for several of our genetic strains. Inhibition of ecdysone secretion by JH occurs only in the last larval instar and we have no independent way of estimating the theoretical maximal size for early instars. Conservatively we assume that in every instar *a = w*_*i*_ *11.*7*, where *w*_*i*_ (the initial mass) is equal to the final mass of the previous instar.

**Fig 6:**
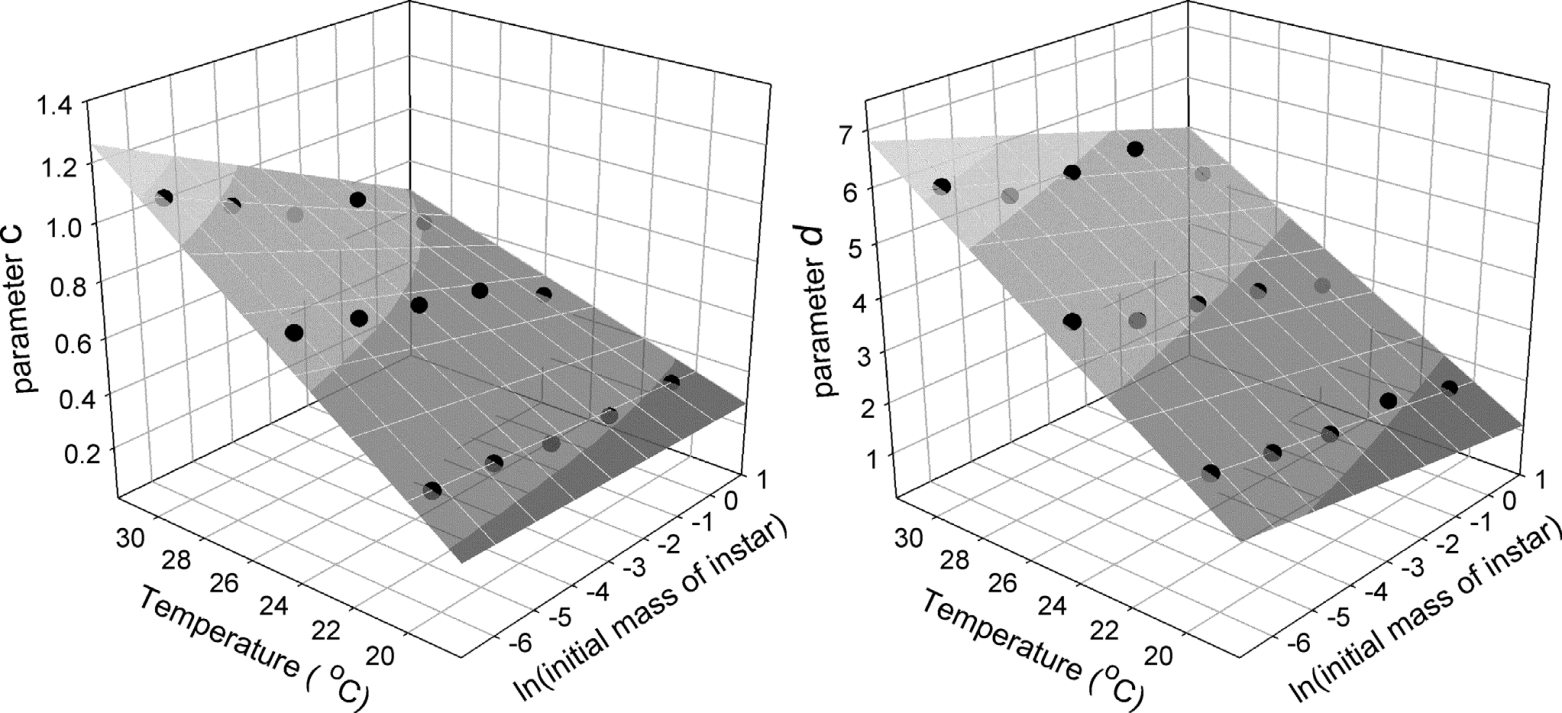
Dependence of Gompertz parameters on initial szie and temperature. Effect of initial size (natural log of mass) and temperature on the values of Gompertz parameters *c* (left panel) and *d* (right panel) in the wild-type strain. The coefficients for the regression are: for parameter *c*, using the natural logarithm of initial mass of an instar, k_1_ = 0.0824, k_2_ = 0.0344, k_3_ = -0.0049, k_4_ = -0.3330, r^2^ = 0.986, p<0.0001; for parameter *d*, k_1_ = -0.0157, k_2_ = 0.2899, k_3_ = -0.0068, k_4_ = -3.9937, r^2^ = 0.985, p<0.0001.

The parameters *a*, *c* and *d* control the shape of the growth trajectory. The parameter *a* is a function of the initial mass of the instar, as noted above. Parameters *c* and *d* depend on the initial size of a larva and by temperature. The joint effects of initial size and temperature were determined by multivariate regression using the model: *k*_1_ * ln(*initaial mass*) + *k*_2_ * *temperature* + *k*_3_ * (ln(*initaial mass*) * *temperature* + *k*_4_. The values of the coefficients and the graphs for the regression are given in Fig 6.

### The cause of the declining growth exponent

The Gompertz equation implies that there is a decline in the growth exponent during the instar. There are three independent processes at work that constrain pure exponential growth and cause the decline in the growth exponent embodied in the Gompertz equation. The first is the limited capacity of the exoskeleton to expand. Although the endocuticle can grow in surface area by intussception [31,32], the epicuticle cannot grow and becomes increasingly stretched as the larva grows. Eventually this reaches a maximum stretch after which growth is no longer possible [33]. The gradually increasing tension exerted by the epicuticle slows the growth rate. The second is the limited capacity of the tracheal system to supply oxygen to a growing body. The size of the tracheal system is set at the beginning of the instar and does not grow during the intermolt period [17]. Thus the capacity for oxygen delivery is constant but the growing body requires increasing amounts of oxygen. The demand for oxygen gradually outruns the capacity of the tracheal system to deliver it and this gradually reduces the growth rate. Finally, if the gut grows hypo-allometrically with respect to body mass its relative capacity to transport nutrients would gradually decrease and this may provide a limiting factor. Whether one of these three processes dominates is not known, since all can gradually constrain further growth as the larva becomes larger.

### The growth rate changes continually

**Fig 7:**
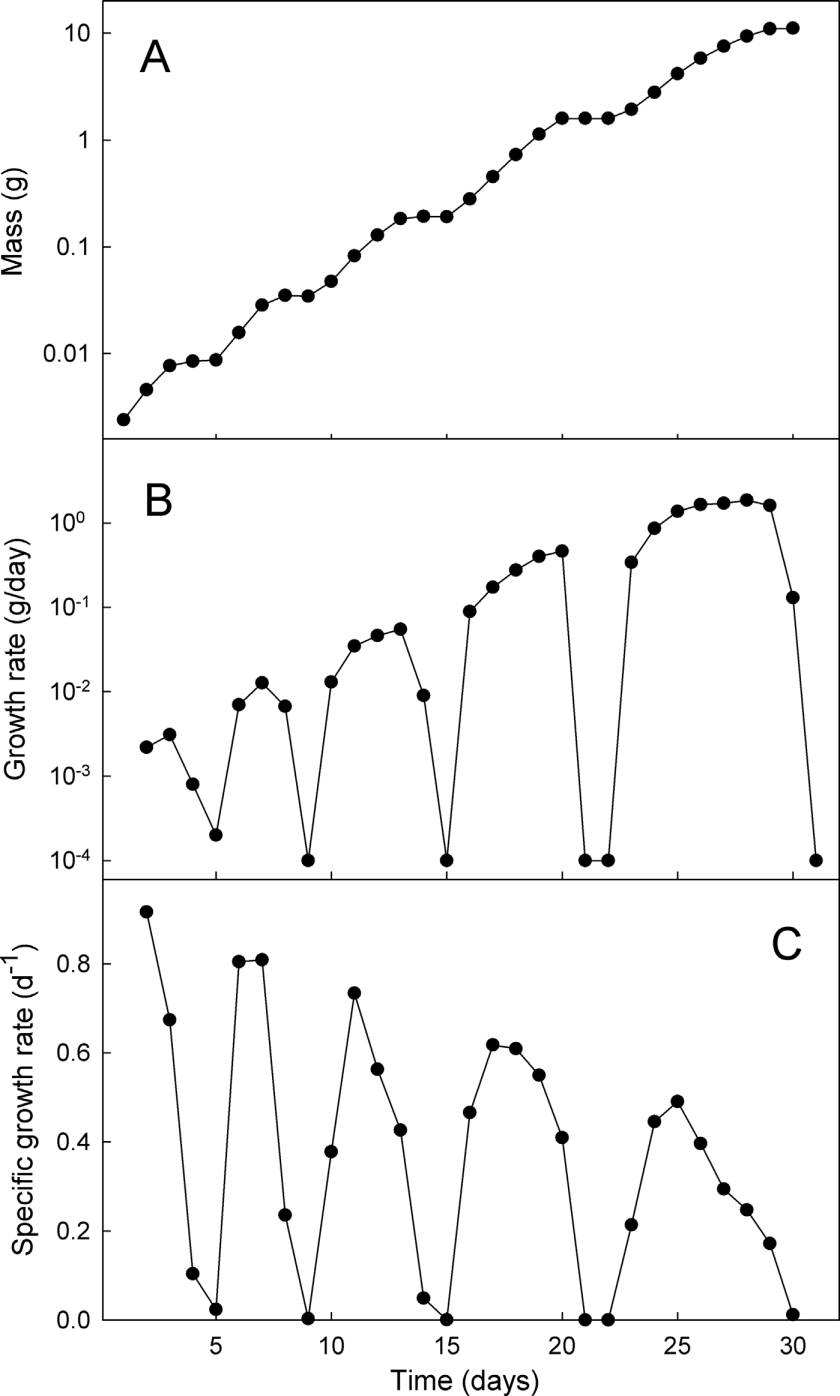
Growth rates change continually. Example of size and the rates of growth throughout larval life of an individual larva at 20°C. (A) growth, (B) growth rate, and (C) mass-specific growth rate.

After a molt larval growth rate gradually increases to a maximum and then declines as the larva prepares for the next molt. The growth rate of a typical wild-type *Manduca* is shown in Fig 7. The growth rate changes from day to day so it is difficult to define a characteristic “rate” either within an instar or across the entire growth trajectory. Useful measures for comparisons among growth trajectories are the mean growth rate of an instar (weight increase divided by number of days between molts), the peak growth rate (at the inflection point of the growth trajectory in each instar), and the mass-specific mean growth rates and peak growth rates. We will use each of these measures in the analyses below.

### The mass-specific growth rates decline from instar to instar

**Fig 8:**
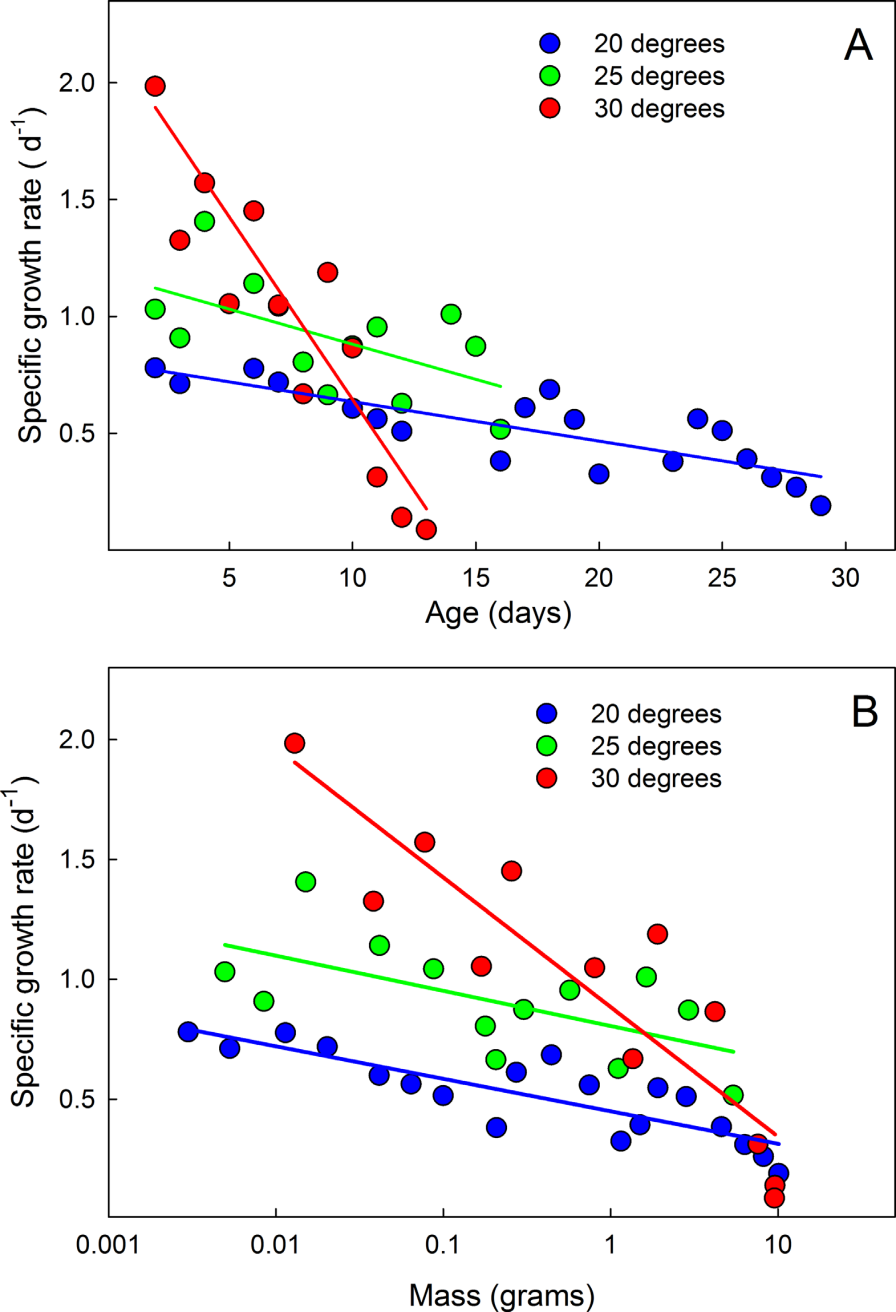
Mean mass-specific growth rates decline during larval life. (A) Declining specific growth rate with time. (B) Declining specific growth rate with mass. Each point is the mean value on successive days of 14 larvae at 20°C, and 15 larvae at 25 and 30°C. Larvae that were molting (and thus not growing) on a given day were omitted from the calculations.

The overall growth trajectory during the entire larval life is not quite exponential (Fig 7A). In addition to the brief interruptions caused by the molts, the exponential rate of growth appears to decline from instar to instar (i.e. growth is not linear when plotted on semilogarithmic axes). This is illustrated by examining the mass-dependent growth rate over time. If the growth rate increased exponentially from instar to instar, the mass-specific growth rate would be the same in each successive instar. Instead, both the mean (Fig 8) and the maximum specific growth rate (Fig 7C) in each instar decrease throughout larval life. The specific growth rate is higher at high temperatures, but converge at approximately the same value at the end of the growth phase (Fig 8).

### Very slow-growing larvae molt on a time schedule

**Fig 9:**
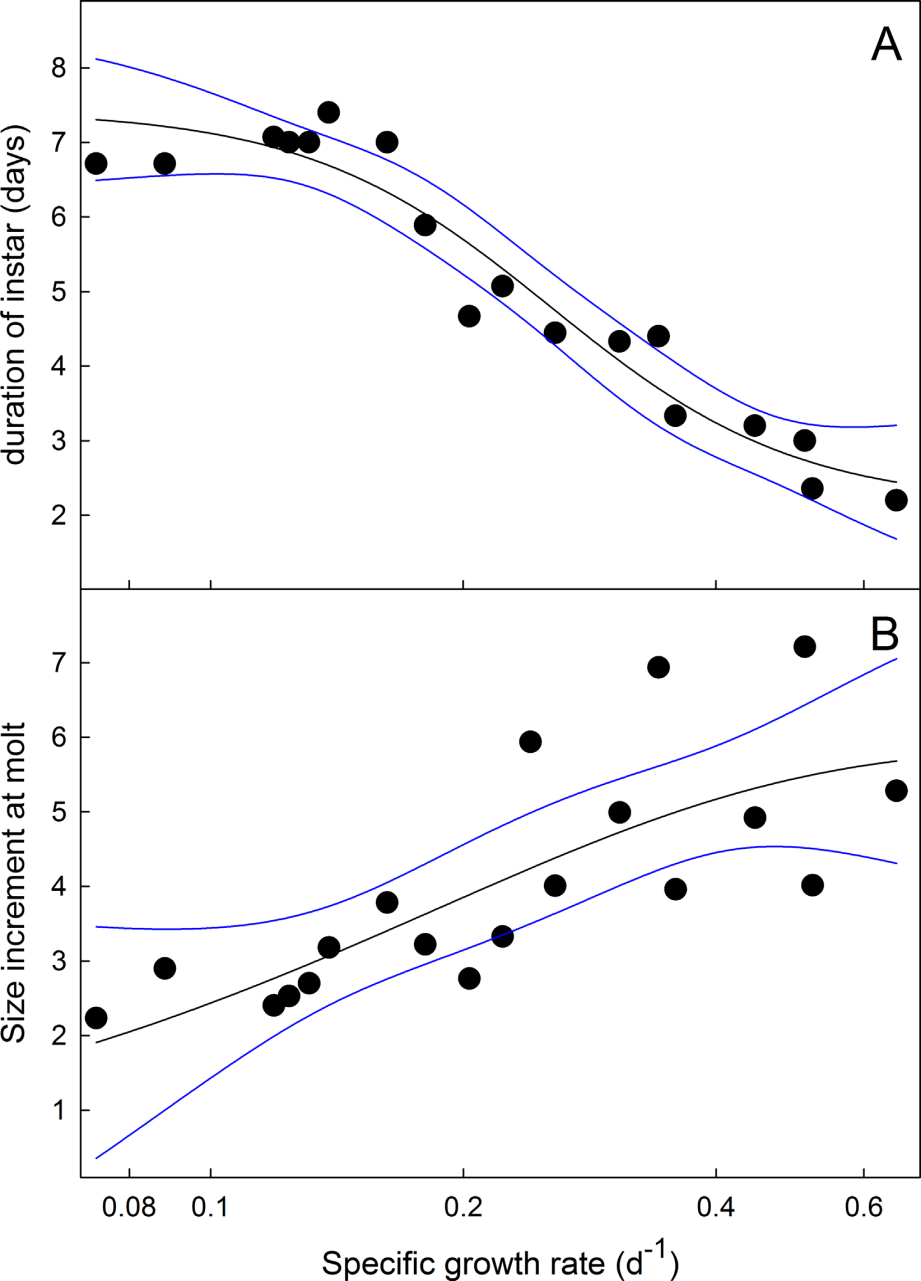
Dependence of duration of instar and size increments at molting on the growth rate. (A) Mean mass-specific growth rate versus duration of instar at 25°C taken from larvae growing on both normal and nutrient-deficient diets. (B) Mean mass specific growth rate versus size increment at each molt. Black lines are fits by non-linear (sigmoidal) regression. Outer bands are 95% confidence intervals.

In slow-growing larvae the operational method for determining the critical weight fails [16]. The critical weight is operationally determined as the weight above which no further feeding is required for a normal time course to metamorphosis. In *Manduca* last instar larvae the critical weight is slightly higher than half the weight to which the larva will eventually grow. When larvae grow slowly due to nutrient restriction, larvae of the same weight (well below the critical weight, as long as they are above the minimum viable weight [34]) have the same time to metamorphosis whether they continue to feed or not [16]. This finding suggest that molting in these larvae may be controlled by a size-independent mechanism [35]. We investigated whether the molts of slow-growing larvae that had supernumerary instars (Fig 3) occurred on a weight-dependent or a time-dependent schedule, that is, are the molts triggered when larvae reach a particular size, or do they occur at regular time intervals. Fig 9 shows the relationship between the mean mass-specific growth rate (mean growth rate during the instar divided by the mid-instar mass) and the length of the instar during the last 3 instars of larvae feeding on low-nutrient diets (60% and 50%). As the specific growth decreases, the duration of the instar increases with a plateau at about 7 days (Fig 9A). The size increment at each molt, by contrast, increases with increasing specific growth rate (Fig 9B). These observations suggest that when larvae grow slowly, the timing of a molt is not determined by a size-dependent mechanism, as it is in normally-growing larvae, as discussed in [35]. Rather, molts occur on a time schedule, after approximately 7 days. As a consequence, slow-growing larvae molt to the next instar and smaller relative weights and can thus require more molts to reach the threshold size.

### A mathematical model for growth and size regulation

The empirical data on growth dynamics outlined above were used to develop a mathematical model for larval growth and body size regulation. The model allows for variation in nutrition and temperature and predicts the duration of each instar, the size at each molt, the number of instars, the final size of the larva, total larval development time and their dependencies on temperature and nutrition. The complete MatLab code for the model is in Supporting Information (S1 File), and the structure of the program is outlined briefly below.

**Fig 10:**
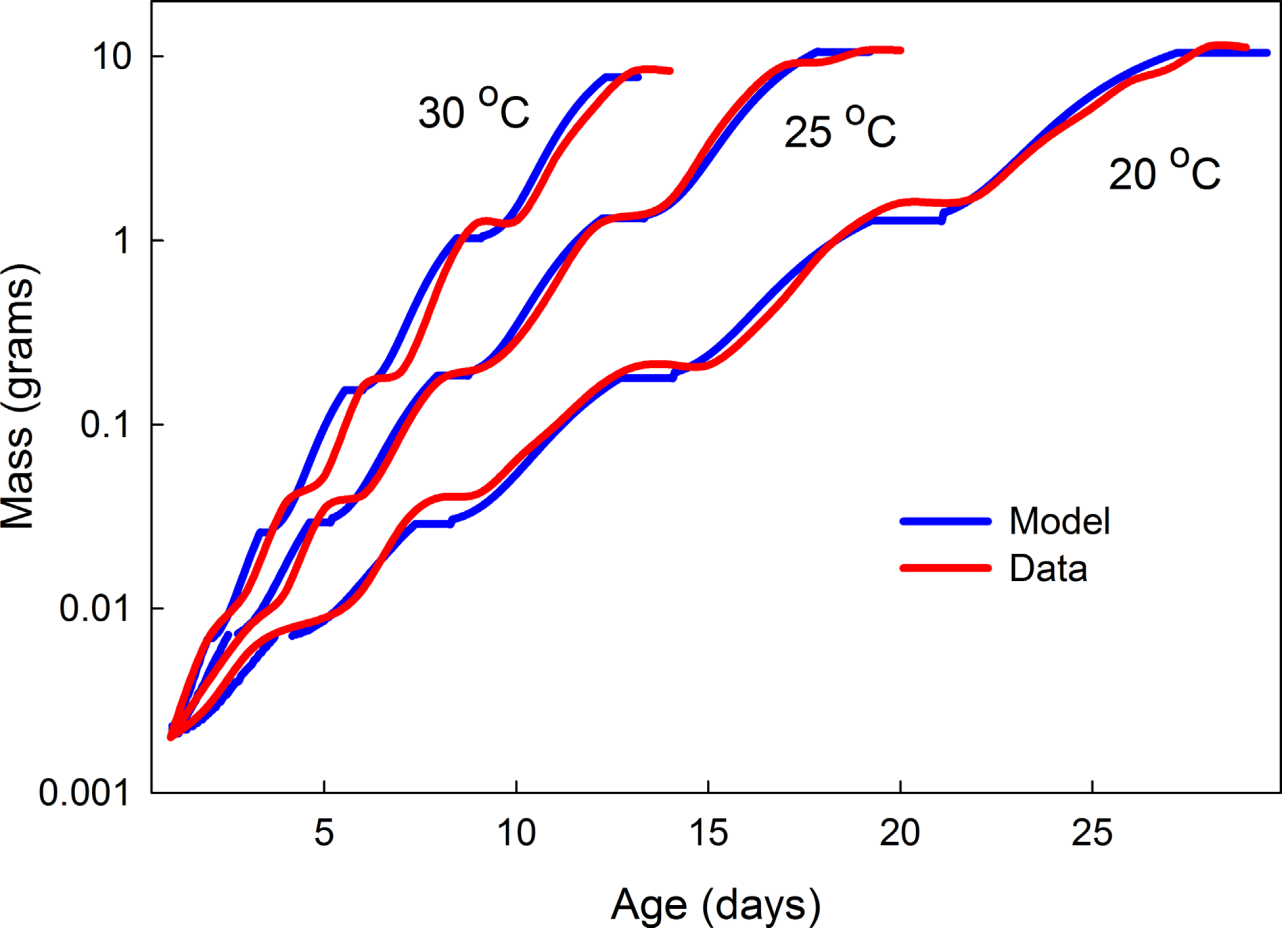
Comparison of model simulations and empirical data. Growth trajectories at three different temperatures (red) and model simulations at those temperatures (blue).

Larvae grow according to a Gompertz trajectory. The value of parameter *a* is calculated as 11.7 times the initial weight. The values of parameters *c* and *d* depend on both initial size of the instar and temperature (Fig 6). After a larva passes the critical weight an interval timer is started that counts down to simulate the duration of the terminal growth phase. The size of the larva at the end of that interval becomes the initial size for the next instar. Growth resumes after a delay that allows for the molt to occur. The duration of this delay depends on temperature and instar. If the initial size of an instar is above threshold size there are no additional instars and final size is the mass achieved at the end of that instar. In the model, temperature affects parameters *c* and *d* and the duration of the terminal growth phase, and the time required for a molt; nutrition only affects parameters *c* and *d*. Fig 10 illustrates the model output and its match to real growth trajectories at 20, 25 and 30 degrees Celsius.

**Fig 11:**
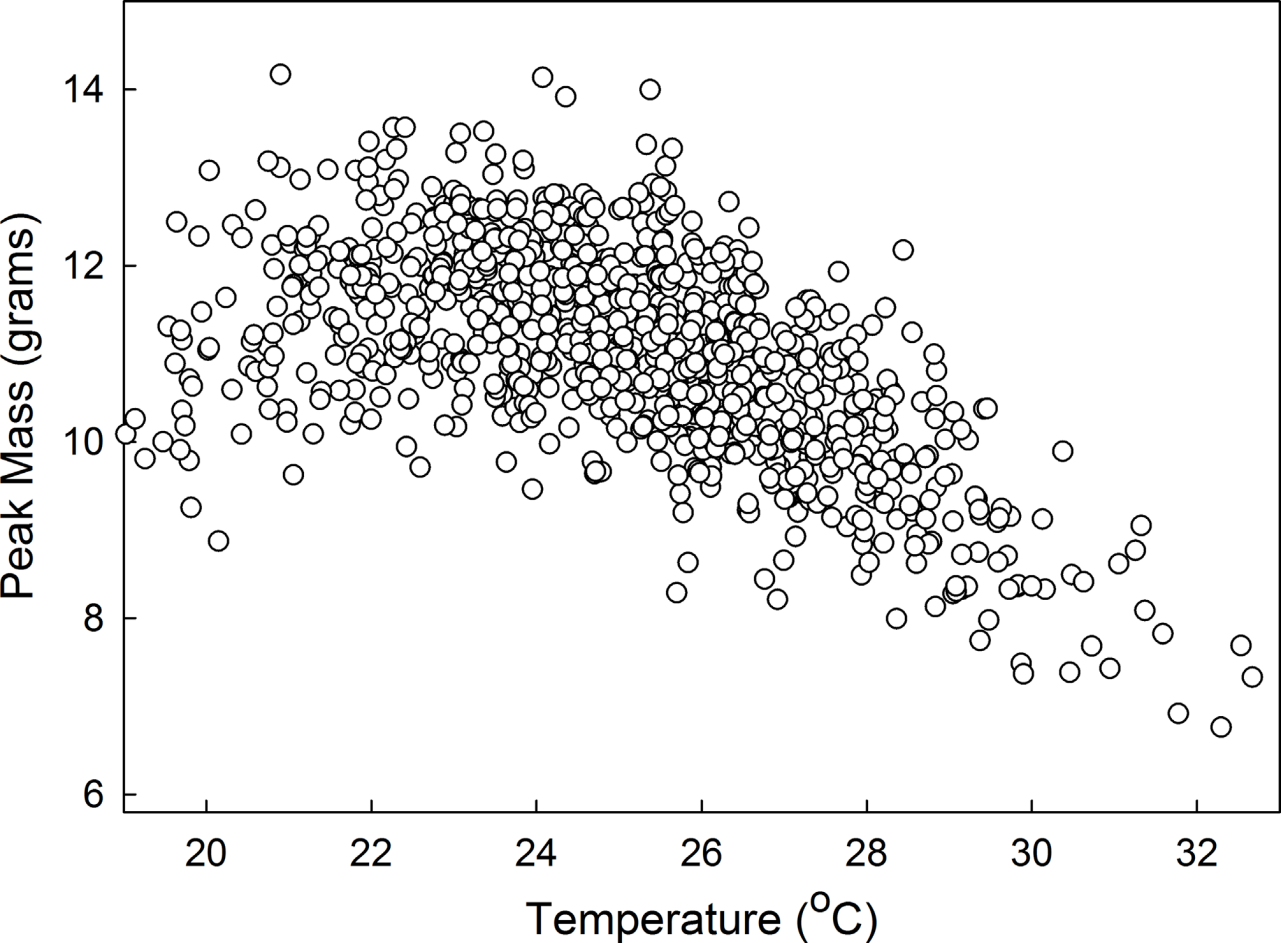
Inverse size-temperature relationship. Results of simulation with the mathematical model for a population of 1000 virtual individuals.

In nature, no two individuals have identical growth trajectories. This can be due to genetic, environmental and stochastic variation in the developmental, physiological and molecular mechanisms that control growth and size assessment. To simulate such variation we developed a population version of the growth model in which the parameters *a*, *c* and *d*, the critical weights and threshold sizes are subject to stochastic variation. Variation is randomly sampled from normal distributions centered on the mean values of each of the parameters, with a standard deviation of 0.02*mean. An individual growth trajectory is generated and the parameters are randomized again for the next growth trajectory. This program produces a population of virtual individuals with distribution of body sizes and development times that closely resemble those of our laboratory populations at different temperatures and nutrient conditions. The means of and relationships among various parameters produced by this model are shown in Table 5, together with corresponding empirical data. The correspondence between empirical data and model data using the amount of stochastic variation outlined above is reasonably good. The model generates the inverse temperature-size relationship (Fig 11) also observed in nature and in the laboratory [36,37]. The values of the correlations among parameters will, of course, depend on the amount of stochastic variation.

**Table 5:** Comparison of model and data correlations among some factors.

	Correlation (95% confidence interval)	
Factors	Data (n = 42)	Model (n = 1000)
mass/time	0.47 (0.29–0.62)	0.45 (0.40–0.50)
time/temperature	-0.97 (-0.98 - -0.96)	-0.97 (-0.973 - -0.967)
mass/temperature	-0.41 (-0.22 - -0.57)	-0.57 (-0.61 - -0.53)
mass/parameter c	0.19 (-0.02–0.38)	0.49 (0.44–0.53)
time/parameter c	-0.43 (-0.25 - -0.58)	-0.47 (-0.54 - -0.41)

## Discussion

Our study of the kinetics of growth of *Manduca* larvae has revealed several interesting and heretofore unexpected features. The first is that the log-transform that is typically used to plot the exponential growth and the growth increment from molt to molt encapsulated in Dyar’s Rule, obscures an interesting underlying pattern. Dyar’s Rule suggests that the growth increment should be constant for every instar. Instead, we found a systematic violation of Dyar’s Rule in that the growth increment increases from instar to instar. This increase is difficult to see in a semilogarithmic plot; exponential regressions actually give very good fits to the data, with r^2^ values that are typically >0.99 (e.g. Fig 1), which is almost certainly why such regressions are accepted as accurate representations of the structure of the data.

Our finding that the size increment actually increases from molt to molt indicates that the mechanism that controls the size and timing of the molt changes systematically as the animal grows. If the size at which each molt occurs is triggered by the size at which oxygen supply by the tracheal system becomes insufficient [17], this implies that the tracheal system increases hyper-allometrically with body mass at each molt. This in turn implies that the critical weight increases in successive larval instars. We previously concluded that the critical weight was a constant multiple of the initial weight in every instar [17], but that conclusion was based on nonlinear regression and thus possibly subject to the same error of interpretation as outlined above for Dyar’s Rule. These findings suggest a cautionary note about using log transforms of size data or exponential regression to study the form of a growth curve: these transforms can camouflage interesting and possibly biologically significant deviations from the perfect exponential trajectory.

Within an instar growth follows Gompertz kinetics. Gompertz growth is initially exponential but the exponent gradually declines to zero, so the overall size curve is a sigmoid. Growth stops when the larva molts, but that occurs well before the asymptote of the Gompertz trajectory is reached. Because of these growth kinetics there is no such thing as a characteristic growth “rate” in each instar. The growth rate changes continuously (Fig 7). The inflection point of the growth trajectory (the maximal growth rate) corresponds to the critical weight. As a larva passes the critical weight the growth rate begins to decline. The decline of the growth rate can be caused by two different biological factors. First, the tracheal system poses a constraint on the growth rate. The tracheal system does not grow during an instar even as mass increases, so the tracheal system becomes progressively less able to supply the ever increasing body mass. Second, the epicuticle cannot grow. The epicuticle is wrinkled at the beginning of the instar and as the larva grows it gradually provides an increasing resistance to enlargement of the body wall. The degree to which each of these limiting factors is responsible for the declining growth exponent is not known.

The molt is initiated at the inflection point of the Gompertz trajectory, when the growth rate is maximal. The larva clearly can continue to grow beyond this point but the rate of growth diminishes and, if oxygen limitation is the constraining factor, an increasing amount of nutrient input will go into metabolism rather than growth.

Oxygen limitation and cuticular stress set physical constraints on the size increment that can be achieved in each instar. The size increment can evolve under selection, as suggested by our data in Fig 2, and by the fact that there is a general trend in insect evolution of a decrease in instar number and increasing size increments at each molt [15]. But in order to maintain an optimal growth rate the oxygen delivery capacity of the tracheal system would also have to evolve, either by increasing its relative volume or decreasing the diffusion distance by means of ventilatory structures like air sacs.

The number of larval instars in *Manduca* is indeterminate, as it is in most insects [19,38], in spite of the fact that in *Manduca* it seldom differs from five. We show that by manipulation of nutrition and temperature *Manduca* can be made to go through as many as eight larval instars. The critical parameter that determines instar number is the molt at which a larva surpasses the threshold size [19,20]. Larva that molt above threshold size enter the last larval instar, those below do not. Thus if larvae grow by small increment at each molt it takes more molts to reach threshold size than if they grow by large increments. Threshold size is a genetically-determined parameter. At the population level the threshold size has a mean and a distribution that can evolve under selection. Selection on body size resulted not only in a change in the mean threshold size but also in a marked decrease of variation around the mean (Fig 4). Interestingly, the threshold sizes for the Dwarf and Giant strains were at the ends but within the range of the wild-type. The physiological mechanism of the threshold size is not understood at present.

In many insects the overall larval growth curve, disregarding the periods during a molt when no growth takes place, deviates significantly from the generally-assumed exponential growth [39]. In *Manduca* the apparent exponent declines from instar to instar (Fig 7), supporting previous findings that although the growth rate increases from instar to instar, it increases at a decreasing rate [10,40]. If growth were exponential then the mass-specific growth rate would be constant at all sizes. We found that the specific growth rate declined as the larvae grew larger (Fig 8) and that the decline was more rapid at higher temperatures. Although at high temperatures the specific growth rate is initially higher than it is at low temperatures, the specific growth rates tend to converge to a common (low) value at all temperatures by the time the larva is fully grown.

The specific growth rate is a measure of assimilation [41]. If the entire mass of the organism is equally metabolically active at all sizes and times, then the specific growth rate will be constant. A declining specific growth rate with body size implies that an increasing fraction of the larva is made up of lower-metabolizing tissues. This could be an increasing fraction of fat body, or gut content, or cuticle, and probably a combination of all three. The decreasing specific growth rate is coupled with the decline of the apparent growth exponent with increasing body size (Fig 7).

The mathematical model we developed can be used to study the causal relationships between body size, development time, nutrition, temperature, the critical weight and threshold size. The correlations among the various parameters and outputs of the model depend on the amount of variation that is introduced, so the model can be used as an experimental tool to investigate what kind of variation results in stronger or weaker associations, and, in the case of negative correlations, how tradeoffs between various factors arise and how variation in environmental factors like temperature and nutrition can affect those tradeoffs [42,43].

## Conclusions

Our analysis of the growth kinetics of *Manduca* has revealed several interesting and some surprising features of the regulation of body size and development time that are likely to be widespread among insects, and that raise a number of questions for future research. One question is what causes the progressive increase in the size increment at each molt? The observation implies that the mechanism that triggers a molt scales allometrically with body size. In *Manduca* the molt is triggered by an oxygen insufficiency imposed by a tracheal system that only grows when a larva molts but stays constant during the intermolt period when somatic growth takes place [17]. Perhaps the mean length or the volume of the tracheal system scales allometrically with body mass. Another question is what causes the decline in the specific growth rate, and why does it converge to approximately the same low value at all temperatures? Perhaps a fully grown larva has a characteristic mix of tissues with different metabolic rates that is independent of previous growth.

What controls development time? From a physiological perspective development time is an emergent property. This is because molting and metamorphosis are triggered by size, not by time. Fast growing larvae simply take less time to reach the species-characteristic size than do slow growing ones. Thus the only ways to alter development time is to alter the growth rate or the size-assessment mechanism. Both the critical weight, which controls the size at which each molt will occur, and the threshold size, which controls when metamorphosis will occur, can be altered by selection on body size or development time (this paper and [21]). But we showed here that in very slow-growing larvae the size-dependent mechanism does not operate (see also [17,35]), but larvae molt on a time schedule. This could be a safety mechanism that allows a larva to grow and molt under nutritional stress, much like the bail-out response of *Drosophila* [44]. Non-size-dependent molts occur after about 7 days. Since a molt is stimulated by secretion of the molting hormone, ecdysone [45], this raises the question of how time-dependent ecdysone secretion is controlled.

Perhaps the most intriguing question is what constitutes the threshold size? Threshold size is the ultimate cause of body size, because it determines, indirectly, at what size growth will stop. We define threshold size operationally as the size (which can be measured as mass or head capsule width [19,20]) at the beginning of an instar that determines whether the larva is in the last instar. But this operational way of finding threshold size says nothing about what exactly the larva itself is measuring that correlates so well with mass. The intriguing problem is that threshold size, unlike the critical weight, is not, as far as we can determine, a relative measure. Threshold size in effect determines the species-characteristic body size, which is an absolute measure. Selection on body size can alter threshold size (Fig 4), so there is genetic variability in the mechanism that controls threshold. The comparative study of genetic strains with different threshold sizes may allow us to unravel the molecular and physiological mechanisms of threshold size.

## Supporting Information

S1 File A mathematical model for growth, size and time at metamorphosis of *Manduca sexta*, implemented in MatLab. (ZIP)
